# MTOR inhibition reversed drug resistance after combination radiation with erlotinib in lung adenocarcinoma

**DOI:** 10.18632/oncotarget.12423

**Published:** 2016-10-04

**Authors:** Hongqing Zhuang, Jing Bai, Joe Y. Chang, Zhiyong Yuan, Ping Wang

**Affiliations:** ^1^ Department of Radiotherapy, Tianjin Medical University Cancer Institute and Hospital, National Clinical Research Center for Cancer, Tianjin Key Laboratory of Cancer Prevention and Therapy, and Tianjin Lung Cancer Center, Tianjin, China; ^2^ Department of Radiotherapy, Baotou Cancer Hospital, Neimenggu, China; ^3^ Department of Radiation Oncology, Division of Radiation Oncology, the University of Texas MD Anderson Cancer Center, Houston, TX, USA

**Keywords:** erlotinib, radiation, drug resistance, everolimus, mTOR

## Abstract

**Objective:**

To investigate the effects of mTOR inhibition on drug resistance in lung adenocarcinoma after combined radiation and erlotinib therapy.

**Results:**

Combined radiation and erlotinib therapy produced clear radiosensitization effects both *in vitro* and *in vivo*; however, tumor cells remained drug resistant. Additionally, combined radiation and erlotinib therapy significantly increased p-AKT and p-P70 levels. After mTOR inhibition, the number of surviving cells significantly decreased compared with that before inhibition, and the *in vivo* growth curve was significantly reduced.

**Methods:**

The effects of combined radiation and erlotinib therapy on tumor inhibition and drug resistance were evaluated by *in vitro* survival curves in PC9 lung adenocarcinoma cell line and *in vivo* growth curves in nude mouse xenograft tumor model respectively. The association between tumor drug resistance and the phosphatidylinositol 3-kinase/protein kinase B/mechanistic target of rapamycin (PI3K-AKT-mTOR) pathway was measured by western blot, assessing the changes in protein kinase B (AKT), phosphor-AKT (p-AKT), P70, and p-P70 protein levels. MTOR was inhibited using everolimus, and changes in AKT, p-AKT, P70, and p-P70 levels were observed. Furthermore, changes in *in vitro* survival curves, and *in vivo* growth curves before and after mTOR inhibition were evaluated to confirm its effects on drug resistance in lung adenocarcinoma after combined radiation and TKI therapy.

**Conclusion:**

mTOR was associated with drug resistance in lung adenocarcinoma after radiation combined with TKI, and MTOR inhibition reversed drug resistance in lung adenocarcinoma after combined radiation and TKI therapy.

## INTRODUCTION

Increasing applications for radiotherapy combined with tyrosine kinase inhibitors (TKIs) are evident in clinical practice [1–5], but drug resistance after this combination therapy is common in patients [5–10]. However, current treatment of this drug resistance mostly involves treatment of simple TKI drug resistance [6–10]; the mechanism of this drug resistance after the combined radiation and TKI therapy remains unknown. This study investigated the expression changes in the phosphatidylinositol 3-kinase/protein kinase B/mechanistic target of rapamycin (PI3K-AKT-MTOR) pathway after combined radiation and TKI therapy and this pathway's inhibition to reverse drug resistance, which could provide potential targets and ideas for resolving clinical drug resistance after combined radiotherapy and TKI.

## RESULTS

### Tumor inhibition and drug resistance after combined erlotinib and radiation treatment

**Figure 1:**
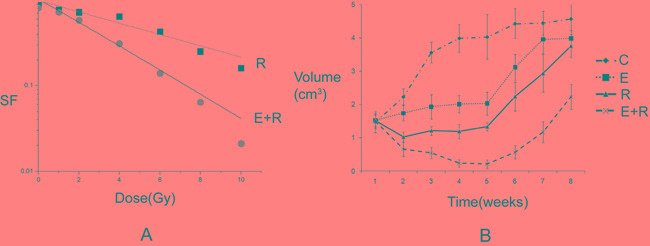
Tumor inhibition and drug resistance after combined erlotinib and radiation treatment **A.** The survival fractions of radiation alone group, and combined radiation and erlotinib group *in vitro*. SER=2.18. P value less than 0.05 of comparison of two curves. But the final surviving fraction was 0.019±0.008, even after combination therapy. **B.** The growth curves of radiation alone group, and combined radiation and erlotinib group *in vivo*. When the curve of combination treatment compared with the other curves, all the P values were less than 0.05. However, after 6 weeks, the tumor volumes increased again even under combination treatment.

By measuring surviving fraction *in vitro* and tumor volumes *in vivo*, erlotinib and radiation both had inhibitory effects on tumor cells. However, after long-term treatment, colony formation survival curves revealed that the final surviving fraction was 0.019±0.008, even after combination therapy. Additional tumor cells were drug-resistant and formed colonies after combined erlotinib and radiation therapy. For xenograft tumors, the combined action of erlotinib and radiation produced a greater inhibitory effect than that of each individual agent. However, tumor volumes still slowly increased after 6 weeks of treatment (SF, and growth curve results are shown in Figure 1). Thus, these results showed that erlotinib combined with radiation had synergistically inhibited tumor growth; however, some cells remained drug resistant.

### Association between drug resistance and PI3K-AKT-mTOR pathway activity

**Figure 2:**
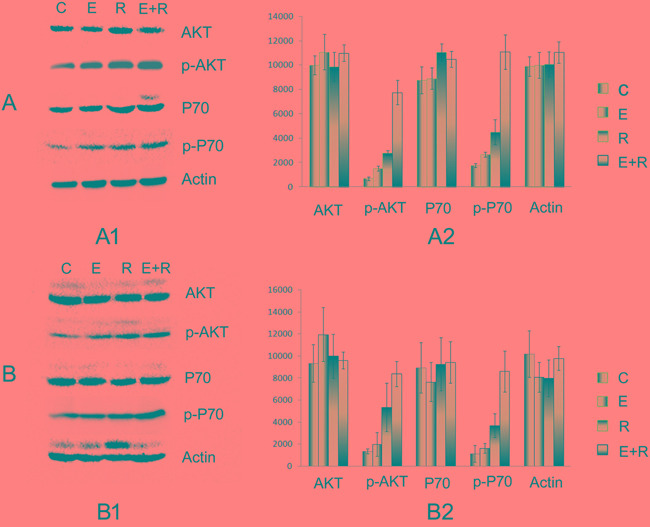
Association between drug resistance and PI3K-AKT-mTOR pathway activity **A.** The AKT, p-AKT, P70, p-P70 expression of PC9 cell line *in vitro*. **A1.** The Western blot electrophoresis of the different treatment groups. **A2.** The Gray value histogram of the AKT, p-AKT, P70, p-P70 expression *in vitro*. **B.** The AKT, p-AKT, P70, p-P70 expression *in vivo*. **B1.** The western blot electrophoresis of the different treatment groups. **B2.** The Gray value histogram of the AKT, p-AKT, P70, p-P70 expression. P-AKT and p-P70 expression significantly increased compared with that of the control group; in addition, p-AKT and p-P70 expression in the combined erlotinib and radiation treatment group was higher than that in the single-agent treatment groups. These results showed that erlotinib and radiation both increased PI3K-AKT-mTOR pathway activity *in vitro* and *in vivo*.

By western blot, changes in total AKT and P70 protein expression between single-agent erlotinib or radiation treatment and combined treatment were not significantly different. However, p-AKT and p-P70 expression significantly increased compared with that in control group; in addition, p-AKT and p-P70 expression in the combined erlotinib and radiation treatment group was higher than that in the single-agent treatment groups (western blot and densitometry measurements are shown in Figure 2). Thus, these results showed that erlotinib and radiation both increased PI3K-AKT-mTOR pathway activity. This pathway activity also increased significantly after combined erlotinib and radiation therapy.

### Changes in PI3K-AKT-mTOR pathway activity after mTOR inhibition with everolimus

**Figure 3:**
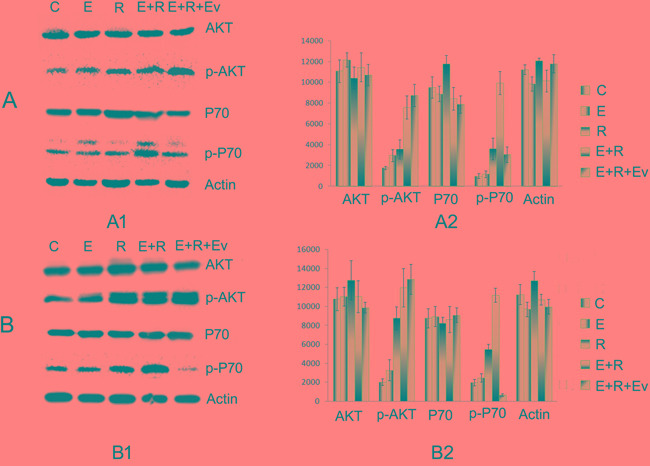
The drug resistance induced by radiation combined with erlotinib was inhibited by everolimus **A.** The AKT, p-AKT, P70, p-P70 expression of PC9 cell line *in vitro*. **A1.** The Western blot electrophoresis of the different treatment groups. **A2.** The Gray value histogram of the AKT, p-AKT, P70, p-P70 expression *in vitro*. **B.** The AKT, p-AKT, P70, p-P70 expression *in vivo*. **B1.** The western blot electrophoresis of the different treatment groups. **B2.** The Gray value histogram of the AKT, p-AKT, P70, p-P70 expression. Western blot showed that protein expression of total AKT and P70 was not significantly altered; however, p-AKT expression slightly increased, and p-P70 expression, both *in vivo* and *in vitro*, significantly decreased in combined radiation, erlotinib and everolimus group compared with those in the combined radiation and erlotinib group.

The drug resistance induced by radiation combined with erlotinib was inhibited by everolimus in tumor cells and xenograft tumors in nude mice. Western blot showed that protein expression of total AKT and P70 was not significantly altered; however, p-AKT expression slightly increased, and p-P70 expression, both *in vivo* and *in vitro*, significantly decreased compared with that in the combined radiation and erlotinib group. These results further confirmed that the PI3K-AKT-mTOR pathway is an important route for the development of drug resistance (Figure 3).

### The effect of everolimus on drug resistance in adenocarcinoma after combined radiation and TKI therapy

**Figure 4:**
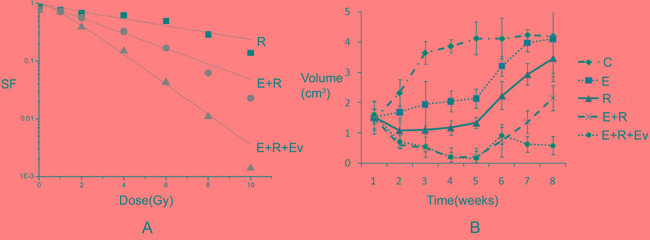
The effect of everolimus on drug resistance in adenocarcinoma after combined radiation and TKI therapy **A.** The survival fraction of different treatment groups *in vitro*. SER_E+R vs R_=1.95, SER _E+R+Ev vs E+R_=2.27. The final surviving fraction of 10Gy dose was 0.008±0.003 at E+R+Ev group. P value less than 0.05 of comparison of each groups. **B.** The growth curve of different treatment groups *in vivo*. Everolimus treatment at week 6 (when tumor xenograft began to progress) significantly inhibited tumor growth, and the growth curve turned downward.

After p-P70S6K expression was reduced by everolimus, further treatment showed that the number of surviving cells significantly decreased compared with that in the combined erlotinib and radiation group. In addition, everolimus treatment at week 6 (when tumor xenograft began to progress) significantly inhibited tumor growth, and the growth curve turned downward (Figure 4). Thus, these results showed that after drug resistance induced by combined radiation and erlotinib treatment occurred in tumors, everolimus was able to reverse this drug resistance.

## DISCUSSION

The PI3K-AKT-mTOR pathway is an important pathway underlying drug resistance induced by combined radiation and TKI therapy, and inhibiting mTOR can reverse this drug resistance in lung adenocarcinoma.

The cross-talk between the EGFR-KRAS-MAPK-ERK and PI3K-AKT-mTOR pathways, the activation of the PI3K-AKT-mTOR pathway by TKI and radiation produce conditions favorable for using everolimus to reverse drug resistance in tumors after combination therapy. First, the EGFR-KRAS-MAPK-ERK pathway can communicate with PI3K through alternative pathways. With the long-term inhibition of EGFR, PI3K-AKT-mTOR pathway activity can be activated through alternative pathways to maintain cell survival, escape TKI attack, and generate secondary drug resistance [16, 17]. In addition, many studies have shown that radiation can increase the activity of the downstream PI3K-AKT-mTOR pathway members through c-MET, which is an important mechanism underlying drug resistance to TKI [18, 19]. With combined TKI and radiation therapy, the PI3K-AKT-mTOR pathway receives double stimulation, and its activity significantly increases, thus producing resistance to radiation and TKI treatment [20–24]. Therefore, the PI3K-AKT-mTOR pathway becomes the interaction point of their action and an important pathway to regulate drug resistance. As an important drug that inhibits mTOR, everolimus decreases mTOR pathway activity, thus reverses drug resistance in tumors.

TKI drug resistance is a longstanding issue; however, drug resistance after radiation combined with TKI should have some similarities and differences from single-agent TKI drug resistance, but no current studies have addressed this question. This study suggested that the PI3K-AKT-mTOR pathway is an important mechanism underlying drug resistance after radiotherapy combined with TKI. Everolimus could reverse this drug resistance induced by combined radiation and TKI therapy in lung adenocarcinoma, which provides an important potential target to treat drug resistance in patients after combination therapy. Indeed, other pathways besides the PI3K-AKT-mTOR pathway could also contribute to drug resistance in tumor cells after combined TKI and radiation. We are currently performing further studies on other possible underlying mechanisms, such as changes in T790M, the effect of radiation on common genes in TKI drug resistance, and the effect of TKI on common genes in radiation resistance.

In summary, radiation is an important mechanism that induces changes in gene expression. Radiotherapy combined with TKI has been extensively applied in clinical practice. Drug resistance in lung adenocarcinoma after combined radiotherapy and TKI should be largely different from that induced by single-agent TKI therapy. This study suggests that the PI3K-AKT-mTOR pathway is an important mechanism underlying drug resistance after combined radiation and TKI therapy and provides ideas and potential targets to reverse this drug resistance. Although additional mechanisms still requiring further study exist, we believe that with continuous in-depth, basic studies and continuous accumulation of clinical practice data, the drug resistance induced by radiotherapy combined with TKI will eventually be resolved.

## MATERIALS AND METHODS

### Agents, cell line and nude mice

RPMI-1640 culture medium was obtained from Gibco (USA, Grand Island), and fetal bovine serum was obtained from Sijiqing Biological Engineering Materials Co., Ltd(Hangzhou, China). Monoclonal antibodies targeting AKT, phosphorylated AKT(p-AKT), P70, phosphorylated P70(p-P70) were purchased from Santa Cruz Biotechnology, Inc. (USA, Dallas, Texas). The CO_2_ incubator used for cell culture was purchased from Heraeus (Germany, Frankfurt), and the high-speed refrigerated centrifuge was also obtained from Heraeus. The flow cytometer was from Beckman Coulter, Inc. (USA, California). The PC9 lung adenocarcinoma cell line with high levels of epidermal growth factor receptor (EGFR) and phospho-EGFR, as reported previously [11, 12] was used in this study. Cells were cultured in RPMI-1640 medium supplemented with 10% fetal bovine serum, 100 IU/ml penicillin, and 100 IU/ml streptomycin in a 37°C incubator with an atmosphere of 5% CO_2_. Cells in the exponential growth phase were irradiated. BALB/c-nu /nu female nude mice of 5-6 weeks were used in *in vivo* experiments. The experiments and feeding were carried out in the SPF condition of the ultra clean laminar flow frame.

### Colony-forming analysis

Colony-forming rates of the tumor cells were determined using the colony formation assay. The experiments on erlotinib-induced radiosensitization included the following treatment groups: control group, radiation alone group, erlotinib alone group, everolimus alone group, combined erlotinib and radiation group, and combined erlotinib and radiation with everolimus group. Cells in the exponential growth phase were trypsinized, counted, diluted, and seeded onto 35-ml flasks. The number of cells seeded onto the flasks was adjusted according to the radiation dose (500, 1000, 2000, 4000, 6000, 8000, and 10000 cells were seeded in 0, 1, 2, 4, 6, 8, and 10Gy groups, respectively). The concentrations of erlotinib and everolimus used were 20 nM. and 10 nM *in vitro*, respectively. A radiation dose of 2Gy/min was selected, and cells were exposed to 0, 1, 2, 4, 6, 8, or 10Gy of radiation after the attachment of cells on the plastic. After 14 days of cell seeding, the culture dishes were collected, and the culture medium was discarded. Cells were fixed and subjected to Giemsa staining. The number of colonies containing more than 50 cells was counted, and the cell survival fraction (SF) was calculated. The single-hit, multi-target model was used to fit the cell survival curves [13]. The experiments were confirmed three times, and each treatment group contained three parallel samples.

### Xenograft analysis

PC9 cells were digested with exponential growth phase, counted and centrifuged at 1 000r/min for 5 min. Cells were suspended and about 1×10^6^ cells was injected into the thigh root of the nude mice. The tumors were observed 3 times a week after inoculation. When the tumor grew to about 1cm of the diameter, the experimental treatment started. The groups were same as the *vitro* experiment, and the radiation doses were same to those *in vitro*. *In vivo* experiment, both erlotinib and everolimus were used in 2mg/kg body weight. Animals' care was in accordance with institution guidelines.

### Western blotting

The expressions of AKT, p-AKT, P70, and p-P70 in the control group, radiation alone group, erlotinib alone group, everolimus alone group, combined erlotinib and radiation group, and combined erlotinib and radiation with everolimus group were examined using Western blotting. The treatments of erlotinib and the everolimus were the same as those described above. Cells were irradiated at a dose of 6Gy. The experimental procedures were performed as follows: 14 days after treatment, the cells were trypsinized and collected. In *in vivo* experiments, the tumors were observed for eight weeks, and then the mice were killed, and the tumors were removed. The total protein was extracted, and the protein concentration was determined by Coomassie brilliant blue staining. The proteins were separated by polyacrylamide gel electrophoresis and transferred onto polyvinylidene difluoride membranes. The membranes were then probed with primary antibodies, washed, incubated with horseradish peroxidase–conjugated secondary antibodies, and washed again. Finally, protein signals were visualized [14, 15].

### Statistical analysis

Origin7.5 software (OriginLab Corporation) was used to fit the cell survival curves. The line charts were drawled with Excel. Data were presented as the mean ± standard deviation and were analyzed using SPSS17.0 software (IBM Corporation). The analysis of student's t test was used to perform comparisons among multiple groups. P values less than 0.05 were considered statistically significant.

## Abbreviations

C: control group
R: radiation group
E+R: combined radiation and erlotinib group
E+R+Ev: combined radiation, erlotinib and everolimus group
